# GC–MS based metabolite profiling, antioxidant and antiurolithiatic properties of apple cider vinegar

**DOI:** 10.2144/fsoa-2023-0035

**Published:** 2023-04-11

**Authors:** Ankul S Singh, Anuragh Singh, Chitra Vellapandian, Radhika Ramaswamy, Margesan Thirumal

**Affiliations:** 1 Department of Pharmacology, SRM College of Pharmacy, SRM Institute of Science and Technology, Kattankulathur, Tamil Nadu, 603203, India; 2 Department of Pharmacognosy, SRM College of Pharmacy, SRM Institute of Science and Technology, Kattankulathur, Tamil Nadu, 603203, India

**Keywords:** antioxidant, antiurolithiatic, apple cider vinegar, DPPH, phytochemical constituents

## Abstract

**Aim:**

To perform *in vitro* analysis of antioxidant and antiurolithiasis to carry out GC–MS-based metabolite profile.

**Materials & methods:**

The effect of apple cider vinegar (ACV) *in vitro*, antioxidant and GC–MS analysis was evaluated. The antioxidant studies were performed. *In vitro* techniques included nucleation, aggregation and growth assay.

**Results & conclusion:**

The presence of polyphenols, flavonoids, alkaloids and carbohydrates was shown. Concentrations from 5–30 μg/ml could dissolve calcium oxalate (p < 0.05) *in vitro*. The IC_50_ value of ACV in DPPH was found to be around 7 μg/ml and the IC_50_ value of the ACV in ABTS assay was around 9 μg/ml. Different phytocompounds were obtained from GC–MS analysis. ACV can be consumed to prevent kidney stones which seems helpful to the current therapy.

## Plain language summary

Due to the modern lifestyle, urolithiasis continues to be a global medical burden and is getting worse every day and seems to have a very high recurrence rate. Urolithiasis can be managed with the pharmacotherapy of drugs and the usage of medicinal plants and nutraceuticals. The effect of apple juice products on urine physiology has been studied profoundly. The product thus obtained was examined for antioxidant assay using DPPH and *in vitro* antiurolithiasis activity. Apple cider vinegar (ACV) exhibited dose-dependent radical scavenging activity against free radicals. The ACV demonstrated a substantial suppression of the DPPH radical in the DPPH assay. ACV could be a potential source of a natural antioxidant drink based on *in vitro*, phytochemical and GC–MS analysis which was noteworthy and thus can be used as a therapeutic agent against bladder stones.

The interplay of several genes with dietary and environmental variables brings about the complex phenotype of kidney stone disease [1]. Although men and people of the White race tend to have urolithiasis more frequently, the recent data point to a more pronounced rise in the prevalence among women and African–Americans [2]. Precision medicine may be made possible by improving our understanding of the polygenic factors influencing the risk of kidney stone disease [3]. Tromethamine-E/acetylcysteine is used for cystine calculi and sodium bicarbonate/ potassium citrate solution for uric acid stones in combination with oral alkalinization [4]. Patients with cystinuria at a lifetime risk for urolithiasis frequently require recurrent procedures and are significantly more likely to experience reduced renal function in the long term [5]. It is advised to start early disease management with a high-liquid intake, pyridoxine and the treatment of any stones with an acute presentation to prevent renal failure and oxalosis crisis [6,7]. Nonetheless, many patients will develop recurrent nephrolithiasis despite advanced medical management and need multiple urologic procedures to ensure free stone status. This arises a need for alternative home remedial treatment to help alleviate the disease and stay healthy without adverse effects [8]. A study established that co-administration of apple cider vinegar (ACV) and a restricted calorie diet positively affected appetite reduction, decreased body weight, BMI, hip circumference and plasma triglyceride levels [9]. ACV is generally known for its antioxidant effects and increased activity of antioxidant enzymes (superoxide dismutase, thiol, catalase and glutathione peroxidase) which might be beneficial in preventing complications [10,11]. ACV has been found to exert antibacterial actions but less susceptible activity against fungi and yeast, which might be beneficial in treating urolithiasis as bacterial infections aid it [12,13]. In Turkey, fruit and vegetables are frequently disinfected in households using traditional surface disinfectants such as ACV and grape vinegar [14]. Through the modulation of the antioxidant defense system, ACV can be helpful for the reduction of obesity induced oxidative stress in high-fat diet rats. It also lowers the risk of obesity related disorders by reducing atherogenic risk [15]. A study also found the beneficial effects of ACV on immunity, growth parameters in *C. auratus* and antifungal activity in *Candida spp.* [16–18]. ACV may aid in the management of Type 2 diabetes and neurological diseases by helping to reduce blood glucose and lipids, weight reduction and hypertension [19]. It makes sense that ACV and *Rosa canina* fruits are used in traditional medicine because they effectively combat the detrimental effects of oxidative stress and counteract the biochemical and histological alterations from H_2_O_2_ [20,21]. The application of 45 ml ACV per day is sought to help normalize fat reserves and body weight without causing any negative side effects [22]. Gallic acid, protocatechuic acid, chlorogenic acid, caffeic acid and p-coumaric acid were the most often found phenolic compounds in fruit vinegar. In contrast, tartaric acid, malic acid, lactic acid, citric acid and succinic acid were the most frequently encountered organic acids. Fruit vinegar can be a useful dietary source of antioxidants since they are often high in polyphenols and organic acids [23]. The research shows that handmade vinegar, which in our case are ones that we make ourselves rather than buying from the market, is of far higher quality than commercial vinegar [24]. We have performed the experiments from self-made vinegar rather than marketed one to perform GC–MS analysis and *in vitro* analysis. GC–MS analysis helps identify the phytochemical constituents, and GC–MS analysis revealed the presence of various phytochemical compounds, which could pave the way for identifying potential compounds and developing novel drugs [25,26]. In this context, our research’s major goal is to screen the apple fruit’s phytochemical composition and assess the antioxidant capacity of ACV. GC–MS analysis of ACV was examined to determine the substances contained in the ACV.

## Materials & methods

### Collection & identification of fruit material

Fresh apple (Malus domestica) was procured from a nearby market. Dr Jayaraman P, Director of the Plant Anatomy Research Center (PARC), Tambaram, validated the specimen of fruit and assigned it the registration no. PARC/2022/3517. The SISCO Research Laboratories (SRL) Pvt. Ltd provided the chemicals required for the investigation.

### Processing & preservation of plant material

The fruit was thoroughly cleaned and rinsed in fresh water. The fruits were then crumbled and thoroughly dried in the shade for over 2 weeks. The dry crumbles were further processed in a blender to a fine grade. It is fundamental to do further research to determine the therapeutic effects by screening the bioactive components of medicinal plants. Maceration was used to extract the substance using ethanol and water in a 70:30 ratio [20]. Whole fruits were chopped into small pieces (seeds were taken out) to obtain homogeneous samples. The fruit pulp was homogenized separately in a blender, weighing around 1 kg. The blended pulp was added with yeast, and the fermentation occurred for a week (Anerobic reaction). The supernatant was filtered with the help of a Whatman filter paper. The filtrate was collected and carried out to perform various tests. Vinegar is made from the conversion of ethyl alcohol to acetic acid by lactic acid bacteria(acetification). A rotary evaporator was used to concentrate and filter the resulting solution. Higher amounts of hydrogen sulfide (H_2_S) in the headspace were produced after fermentation at a lower temperature (18°C). This can be attributed to the higher hydrogen sulfide accumulation due to the prolonged viability of the fermentation media and low fermentation speed [27]. The finalized substance was hygroscopic and kept in a sterile, airtight container for further research.

### Physicochemical analysis

The apple fruit was analyzed to establish their proximate compositions, and sample collection was done to identify various physicochemical parameters of fruit [28] like Moisture content by using 10 ml of apple juice and placing it in a clean and dry crucible, later weighed and dried in an oven at 110°C for 3 h until constant weight. Water activity was measured at 25°C using a water activity meter. Ash content was calculated by burning the juice’s fibers and nutrients at 500°C for 3 h until white ash was produced. A laboratory pH meter measured the pH of 10 ml of apple juice. By titrating 50 ml of dilution (5 ml apple juice diluted with distilled water) with 0.1 N NaOH in the presence of phenolphthalein as an indicator, the titratable acidity of the sample was ascertained. The total soluble solids were calculated using two drops of apple juice on the digital handheld refractometer’s lens. The electrical conductivity of the apple sample was determined using a laboratory conductometer.

### Phytochemical study

A preliminary phytochemical analysis was done to determine whether certain phytochemicals were present or absent. To detect phytoconstituents, multiple qualitative tests were applied using ACV [29]. Phytochemical analysis of nonpolar compounds was carried out by using GC–MS analysis.

### Antioxidant potential of ACV using DPPH assay

The DPPH solution (2,2-diphenyl, 1,1-picrylhydrazyl) was made by combining 1.97 mg of DPPH with 50 ml of ethanol [30]. Using a serial dilution procedure, the ACV was synthesized in various concentrations: 5, 10, 15, 20, 25 and 30 μg/ml. Various concentrations in μg/ml of the sample solution (0.2 ml) were obtained and compared with the industry standard (ascorbic acid). The reaction mixture was vigorously shaken before being allowed to cool for 60 min at room temperature in the dark. The DPPH sample that did not include ACV was treated as a blank. A UV-VIS Shimadzu spectrophotometer was used to measure the absorbance of reaction mixtures at 517 nm [31]. All the results were obtained in triplicates. The formula to calculate the percentage of inhibition was given with the following formula:

$$\%\;\text{Inhibition}=\frac{{\text{A}}_{0}-{\text{A}}_{1}}{{\text{A}}_{0}}\times 100$$

Where, A_0_ = The absorbance of control, A_1_ = The absorbance of sample.

### Antioxidant potential of ACV using ABTS assay

As directed by the Total Antioxidant Ability Assay Kit, the ABTS radical scavenging capacity was assessed (Beyotime Biotechnology Co., Ltd). A 1:1 mixture of the ABTS stock solution and potassium persulfate solution created the ABTS working liquor [32]. Before usage, the mixture remained at room temperature for 12–16 h in the dark. It then took 2–3 days for the mixture to stabilize. The resultant blue-green ABTS radical solution’s concentration was changed to achieve an absorbance of 0.7 0.05 at 734 nm. Using a serial dilution procedure, the ACV was synthesized in various concentrations: 5, 10, 15, 20, 25 and 30 μg/ml. After being incubated at room temperature for 2–6 min, the mixture was measured at 734 nm. All the results were obtained in triplicates. The formula to calculate the percentage of inhibition was given with the following formula:

$$\%\;\text{Inhibition}=\frac{{\text{A}}_{0}-{\text{A}}_{1}}{{\text{A}}_{0}}\times 100$$

Where, A_0_ = The absorbance of control, A_1_ = The absorbance of sample.

### Evaluation of the *in vitro* crystallization assay

According to the approach outlined by Mosquera *et al.*, *in vitro* crystal nucleation and aggregation were done in triplicate [33], and growth assay by Bawari S *et al.* [34].

### Nucleation assay

The effects of ACV on CaOx crystallization were investigated using a nucleation test [35]. The solutions of calcium chloride (0.005 M) and sodium oxalate (0.0075 M) were created in a buffer of Tris-HCL (0.5 M) and NaCl (0.15 M) at pH 6.5. Different dilutions of cystone and ACV (10–100 μg/ml) were created in distilled water. ACV and cystone were diluted, and each dilution was mixed with a solution of calcium chloride and sodium oxalate in equal amounts (3 ml each). These dilutions underwent a 30 min heating process in an oven to 37°C and a cooling process to room temperature. A UV-VIS Shimadzu spectrophotometer was used to determine optical density at 620 nm. The experiment employed a mean value; all data were collected in triplicates.

### Aggregation assay

The effects of ACV on CaOx crystal aggregation were investigated. Separate 0.05 M solutions of sodium oxalate and calcium chloride were made, and then they were combined [36]. This combination underwent an hour-long 60°C water bath heating and an overnight 37°C oven incubation period. CaOx crystal solution (80 mg/100 ml) was made in 0.05 M Tris-HCl and 0.5 M NaCl buffer at pH 6.5 after drying this combination. Different dilutions of cystone and ACV (10–100 mg/ml) were created in distilled water. About 3 ml of the CaOx solution were vortexed with one millimeter of each ACV and cystone dilution. For 30 min, the mixture was incubated at 37°C in the oven. At 620 nm, optical density was captured using a spectrophotometer (UV-VIS Shimadzu). The formula provided for the nucleation assay was used to compute the percentage inhibition of aggregation.

### Growth assay

The development of CaOx crystals was assessed by the technique described by Bawari *et al.* [34], 1.5 ml of a solution containing NaCl (90 mM) buffered with Tris-HCl (10 mM) at pH 7.4 was combined with 4 mM CaCl_2_ solution and 4 mM Na_2_C_2_O_4_ solution, each of which contained 4 mM. 30 ml of CaOx crystal slurry (made at a concentration of 1.5 mg/ml in a buffer containing 50 mM sodium acetate and pH 5.7) were then added to the mixture. The rate of oxalate depletion from the solution was then monitored for 600 s at a wavelength of 214 nm to determine the development of CaOx crystals. Different concentrations of cystone and ACV (10–100 mg/ml) were added to the reaction mixture in distilled water, and the optical density of the mixture was measured using a UV-visible spectrophotometer (UV-VIS Shimadzu).

### GC–MS analysis

An analytical method called GC–MS combines mass spectrometry and GC to identify different chemicals in a test sample. It was done to find specific vital bioactive components in the ACV in contrast to methanol and other polar solvents [37]. Clarus 600 (EI) mass spectrometer linked to a Perkin Elmer Clarus 680 gas chromatograph and running in EI mode at 70 eV with mass charge ratio (*m/z*: 1–1000) were used for the analysis. The instrument was calibrated using a homologous series of n-alkanes. During the chromatographic run, the source temperature was set to 250°C and the quadruple temperature to 150°C. ZB-5MS capillary column of 50 m length, 0.32 mm diameter and 0.25 μm film thickness was used to pack the column to separate the acid components using helium as the carrier gas at a constant flow rate of 1 ml/min. TurboMass version 5.4.2 was the program used to operate the gas chromatograph. NIST-2008’s library version was selected. Conditions for the mass detector were a 2 min delay for the solvent, a temperature of 240°C for the ion source and transfer line, an ionization energy of 70 eV, a scan time of 0.2 s and a scanning interval 0.1 s. With a pH range of 4–6.9, the vinegar sample volume of 1.0 l was injected in split-less mode. The overall running duration of the GC–MS was 35 min. To distinguish between all detected compounds, retention time is defined as the time between elution and injection. The chromatographic spectra of the isolated components acquired after performing GC–MS were compared with the database of spectra typically kept in the GC–MS NIST (2020) library. The device splits the material into parts and vaporizes them for individual component analysis.

### Statistical analysis

The data were analyzed using IBM Corp.’s SPSS version 25 (IBM Corp., NY, USA) and were then put through a one-way analysis of variance utilizing the Duncan multiple range *post hoc* test. Values were presented as the mean ± standard error mean of triplicates and were deemed statistically different at a p < 0.05.

## Results

Urolithiasis can be managed with the pharmacotherapy of drugs and the usage of medicinal plants and nutraceuticals. Daily consumption of specific home remedies and, at the same time, drinking plenty of water and avoiding a sedentary lifestyle can be prophylactic therapy for urolithiasis. The effect of apple juice products on urine physiology has been studied profoundly. The product thus obtained was examined for antioxidant assay using DPPH and *in vitro* antiurolithiasis activity. Physicochemical properties of the Apple fruit were carried out, and observed its characteristic odor, red gold color, 4.14 ± 0.06 pH, 1.17 g/l titratable acidity and showed 85% moisture, 1.85% ash content with 0.85 aw water activity. Electrical conductivity of 0.0135 S/m, total soluble solids of 14.25 ± 0.48 and total acid content of 0.19 ± 0.01%, respectively. The extractive value of ACV was 2.5% from 1 Kg of residual weight. The results of the phytochemical examination also included the presence of phenols, glycosides, alkaloids, phytosterols, tannins and flavonoids.

**Figure 1.**
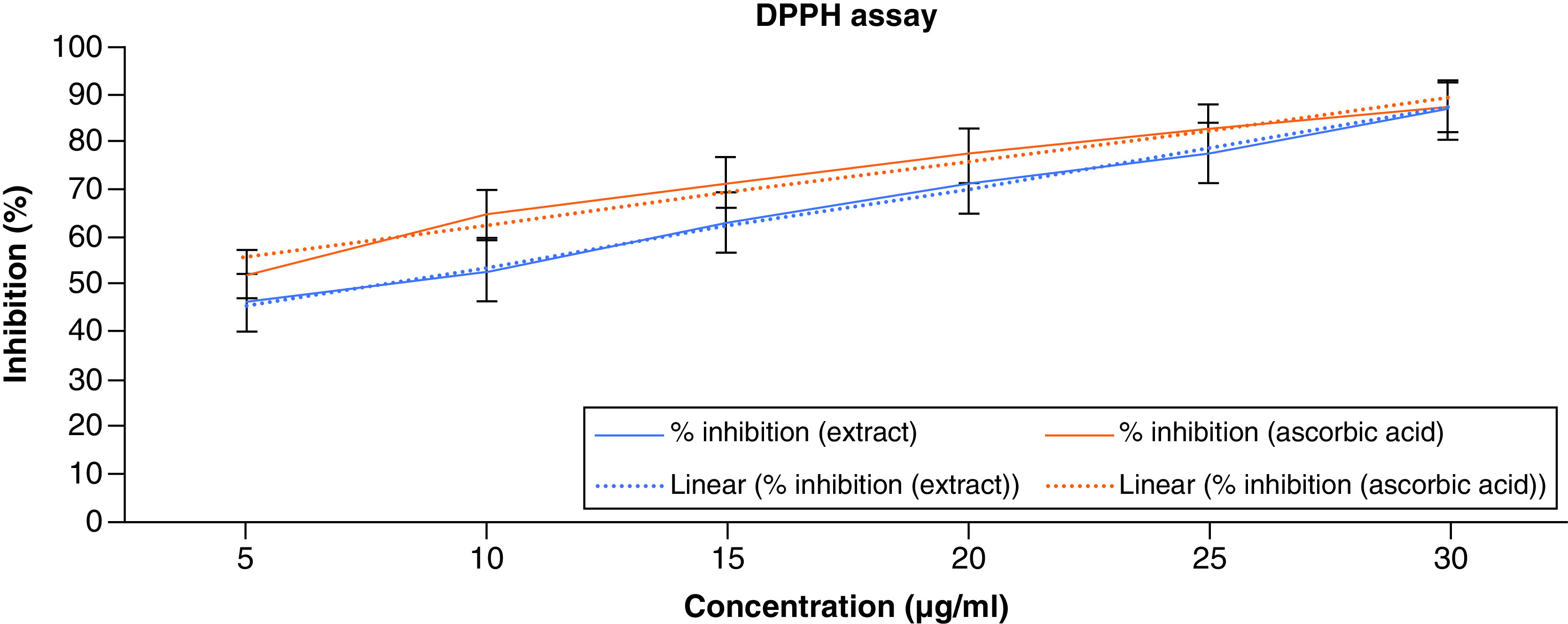
DPPH free radical scavenging activity of apple cider vinegar.

DPPH is a stable free radical at room temperature that can receive an electron or hydrogen radical to transform into a stable diamagnetic molecule. The decrease in DPPH radical absorbance at 517 nm caused by antioxidants measures the radical's capacity for reduction. Since the interaction between antioxidant molecules and radicals intensifies and leads to the radical’s scavenging by hydrogen donation, antioxidants are to blame for the decline in DPPH radical absorption. The color shift from purple to yellow is immediately apparent. As a result, the substrate DPPH is typically utilized to measure antioxidant activity [38]. The findings show that ACV interacts with hydrogen donors in the antioxidant principle and decreases the radicals to match hydrazine. When DPPH radicals interact with appropriate reducing agents, the electrons pair off and the amount of electrons taken up determines how much color is lost in the solution. The DPPH radical scavenging activity of ACV and ascorbic acid is shown in Figure 1. The percentages of DPPH radical scavenging activity of ACV were found to be 46.14, 52.87, 63.12, 70.98 and 86.75% at the concentration of 5–30 μg/ml, respectively. The highest percentage of inhibition was exhibited at 86.75% in the highest concentration. The IC_50_ value of ACV was found to be around 7 μg/ml. The results of the DPPH assay have been illustrated in Figure 1.

**Figure 2.**
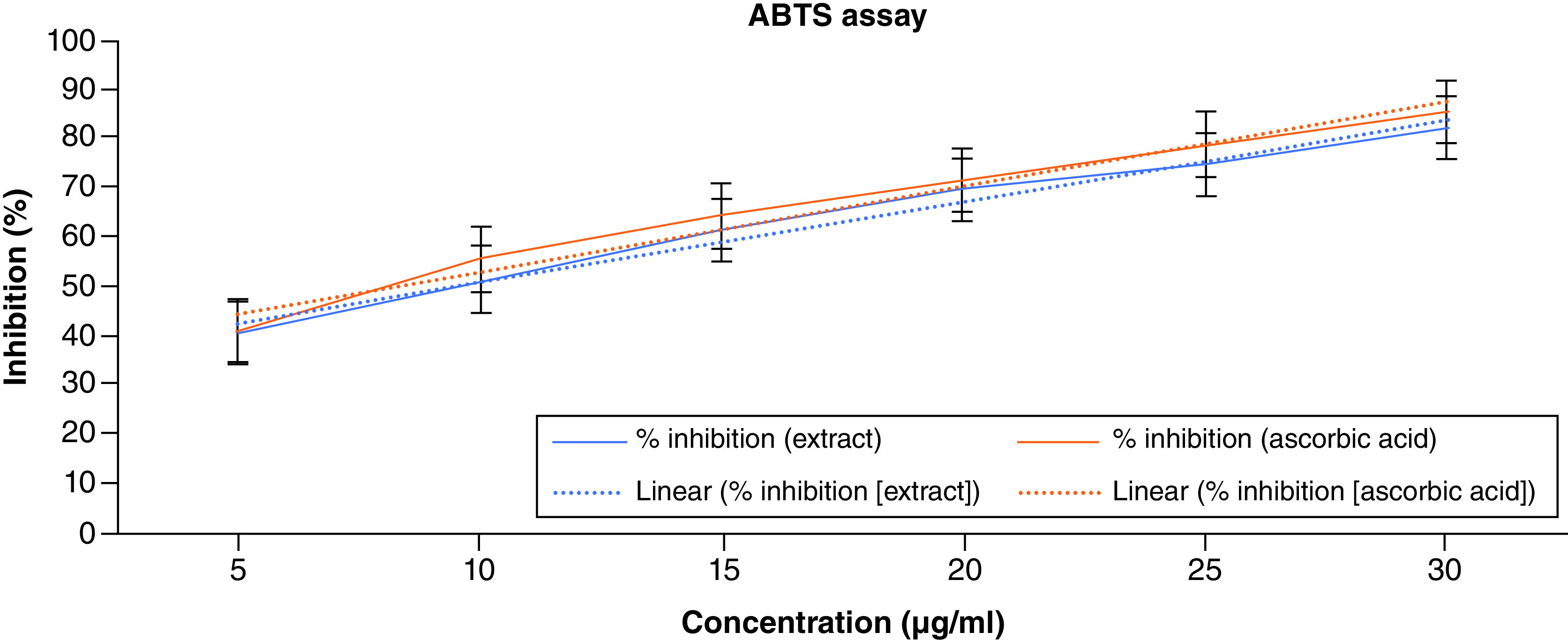
ABTS assay of apple cider vinegar.

ABTS (2,2 azino-bis (3-ethylbenzothiazoline-6-sulphonic) acid is a chemically stable and highly water-soluble substance. It serves as a peroxidase substrate and, upon oxidation, yields a metastable cation. The presence of an antioxidant agent inhibits the spectrophotometric measurement of ABTS4+. The antioxidant compound’s potential increases with decreasing test solution absorption at 734 nm. Both radical quenching and direct reduction via electron transfers can neutralize the ABTS radical (an acid medium facilitates the mechanism by electron transfer) [39]. Although ABTS is relatively stable, it interacts energetically with compounds that may give off hydrogen atoms or electrons, which causes this radical’s blue/green hue to vanish. According to the findings, radicals are reduced to ABTS cations when ACV interacts with hydrogen donors in the antioxidant principle. Figure 2 displays the ability of ACV and ascorbic acid to scavenge ABTS radicals. The percentages of ABTS radical scavenging activity of ACV were found to be 40.14, 51.45, 61.49, 69.35, 74.81 and 82.31% at the concentration of 5–30 μg/ml, respectively. The highest percentage of inhibition was exhibited at 82.31% in the highest concentration. The IC_50_ value of the ACV was around 9 μg/ml. The results of the ABTS assay have been illustrated in Figure 2.

**Figure 3.**
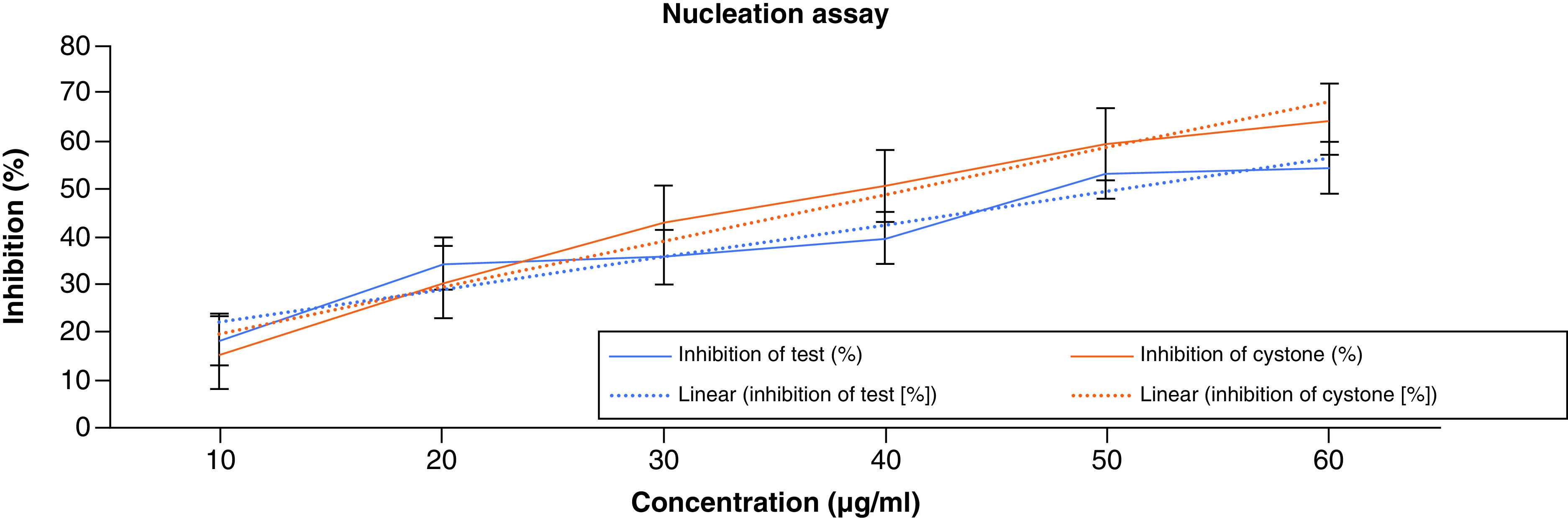
Nucleation assay of apple cider vinegar.

In the nucleation test, the turbidity of the solution was used to quantify the number of crystals produced. The absorbance of the control was deducted from the ACV absorbance in the calculation. With rising ACV concentration, there was a sharp decline in absorbance. The percentage inhibition of the test ranged between 18.49–54.58%, whereas with cystone, it was 15.68–64.45%. The results of the nucleation assay are shown in Figure 3.

### Aggregation assay

**Figure 4.**
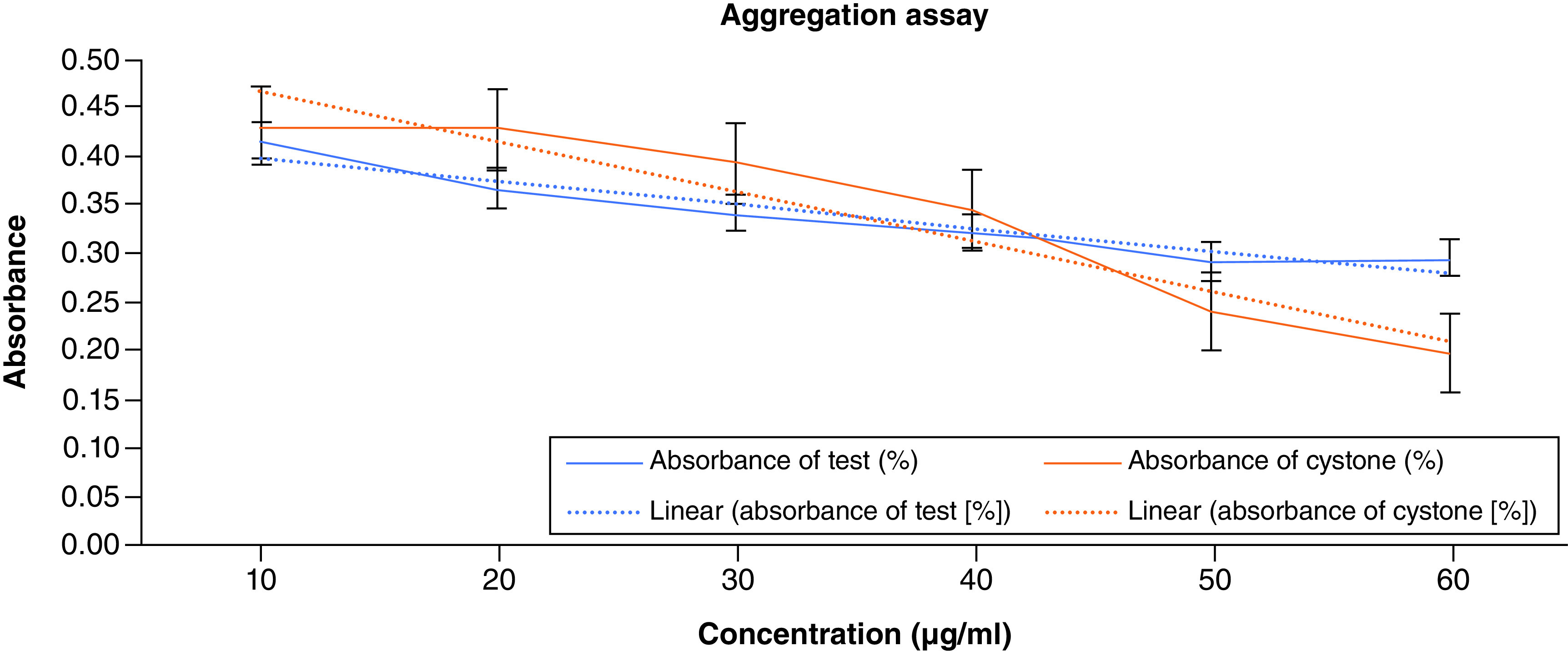
Aggregation assay of apple cider vinegar.

The OD fell as ACV concentration rose, showing that it prevented CaOx particles from aggregating. The absorbance of the test at 10 mg/ml of ACV was found to be 0.415, and the OD was the highest (0.432) of the standard. Higher concentrations were observed to slow down the rate of crystal aggregation. At a dose of 10 mg/ml, the percentage of prevented aggregation for the standard and test samples was determined to be 62.5 and 57.5%, respectively. At the same time, the percentage was at its greatest, 92.27% for the standard and 90.41% for the test, respectively, at the highest concentration. Figure 4 displays the aggregation assay findings.

### Growth assay

**Figure 5.**
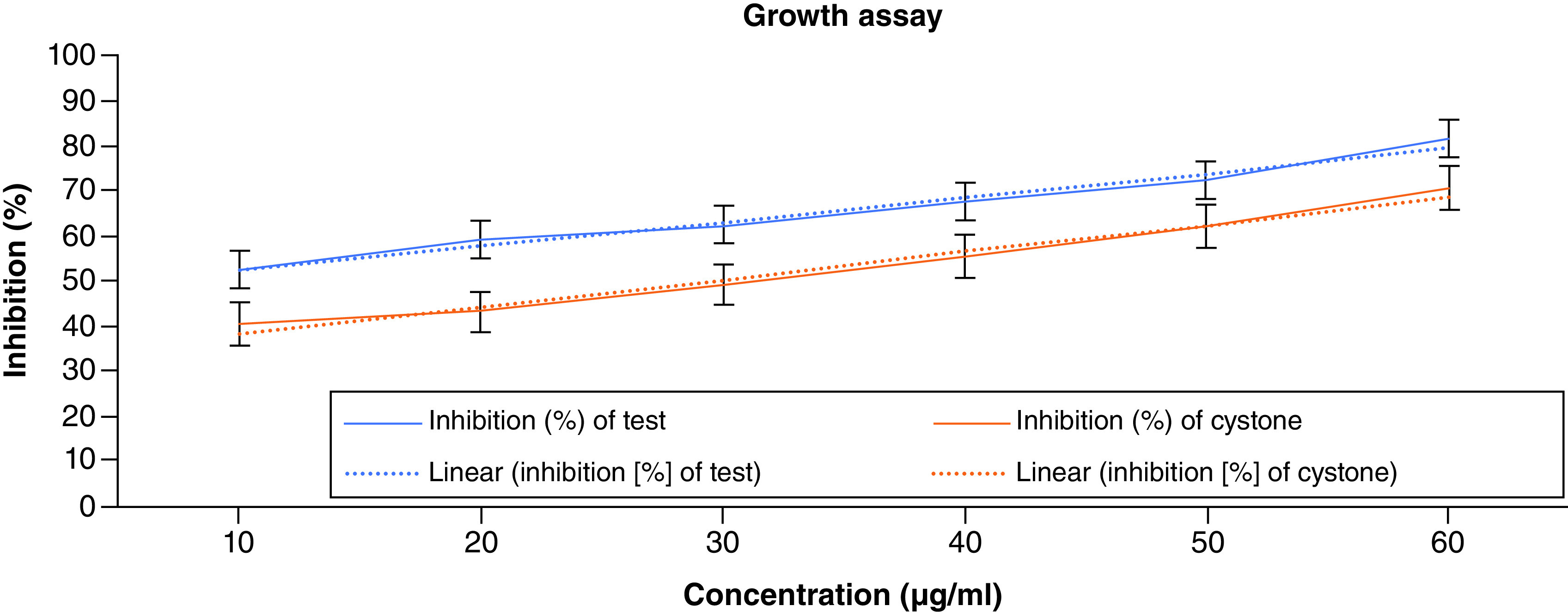
Growth assay of apple cider vinegar.

The growth assay was performed and was correlated with nucleation and aggregation assay, respectively. The absorbance of the standard and test were found out and plotted against the concentration. As the concentration is increased, the absorbance decreases over a period, which specifies the rate of turbidity decreased. This reports that ACV has a potent antiurolithiatic activity as the values of the tests almost matched the standard drug (Cystone). The highest percentage of inhibition was exhibited at the highest concentration. The results of the growth assay are shown in Figure 5.

### GC–MS profiling of ACV

**Figure 6.**
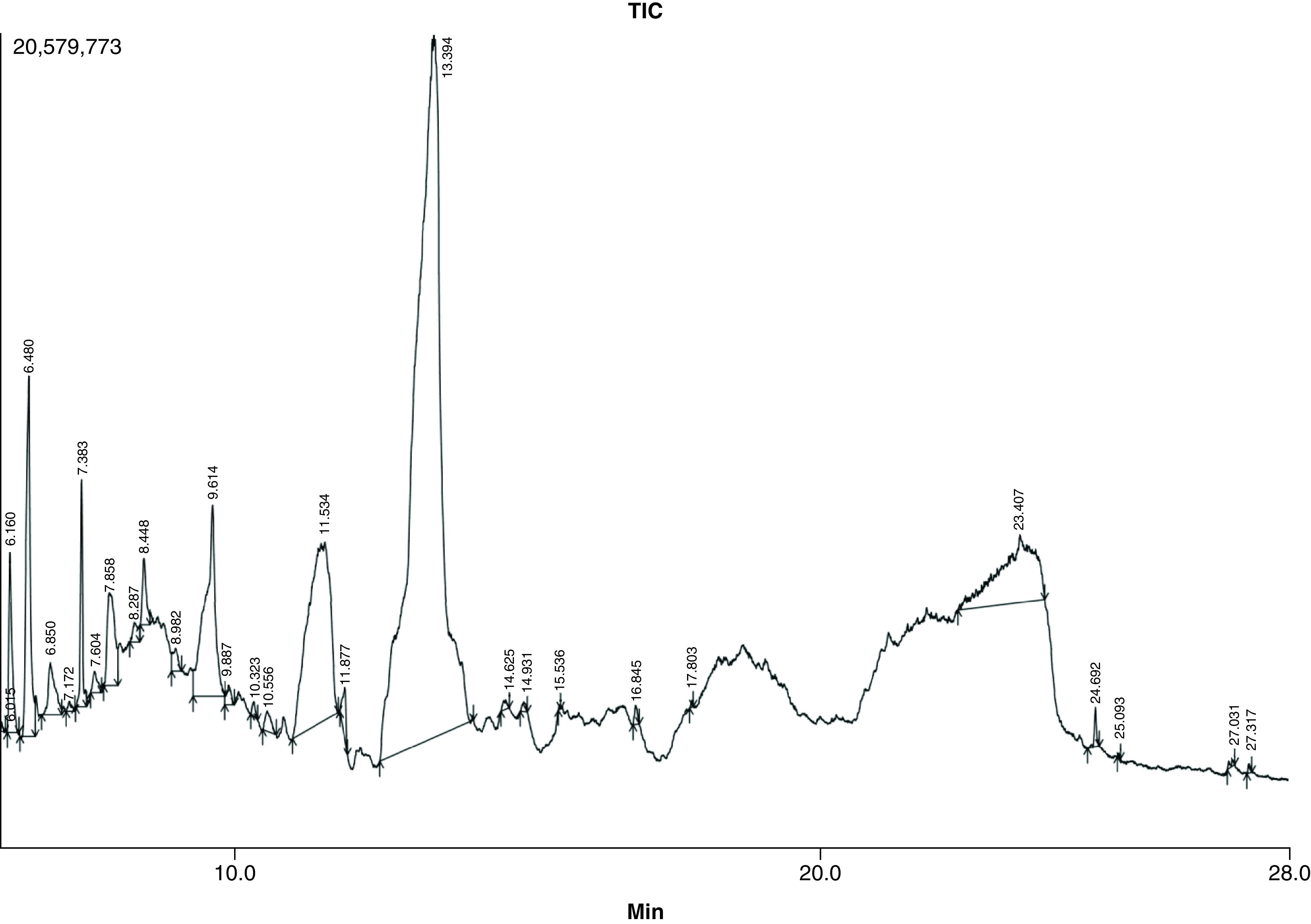
Chromatogram of compounds elucidated from apple cider vinegar using GC–MS analysis.

The chromatogram of the ACV fruit is displayed in Figure 6. Table 1 displays the chromatogram area and height of the chemicals from our analysis that received the most notification. In the ACV solution, 43 compounds were found, accounting for 90% of all collected component peak areas. Five-Hydroxymethylfurfural (49.43%), 1,5-Anhydro-6-Deoxyhexo-2,3-Diulose (10.70%), 3-Deoxy-d-mannoic lactone (6.27%), Bicyclo [2.2.1] Heptane-2-carboxylic acid (5.09%), 1,2-Cyclooctanedione (3.93%), and 2,4-Dihydroxy-2,5-dimethyl-3. The chromatogram demonstrated increased resolution of compounds and greater quantity of the detected phytoconstituents from retention time 10.0–28.0 min. 1,2-Cyclooctanedione compound was observed in RT times 6.480, 2,4-Dihydroxy-2,5-dimethyl-3(2H)-furan-3-one obtained in RT 7.383, Bicyclo [2.2.1] Heptane-2-carboxylic acid Isobutyl amide showed up at 9.614, 1,5-Anhydro-6-Deoxyhexo-2,3-Diulose was observed at 11.534, 5-Hydroxymethylfurfural showed up at 13.394, 3-Deoxy-d-mannoic lactone was observed at 23.407 all showing various biological activities with highest area peak % and height %.

**Table 1.** Top six compounds best notified in GC–MS fingerprint of apple cider vinegar based on activity.

Compound name	R time	Molecular formula	Molecular weight	Area peak, %	Height, %	Biological activity	Ref.
1,2-Cyclooctanedione	6.480	C_8_H_12_O_2_	140.182	3.93	9.14	Antimicrobial	[40]
2,4-Dihydroxy-2,5-dimethyl-3(2H)-furan-3-one	7.383	C_6_H_8_O_4_	144.125	1.27	5.79	Antibacterial, antifungal	[41]
Bicyclo [2.2.1] Heptane-2-carboxylic acid Isobutyl amide	9.614	C_9_H_14_O_2_	154.206	5.09	4.89	Antibacterial, antifungal	[49]
1,5-Anhydro-6-Deoxyhexo-2,3-Diulose	11.534	C_6_H_8_O_4_	144.125	10.70	4.55	Breast cancer, antibacterial, antimicrobial, antioxidant	[50,51]
5-Hydroxymethylfurfural	13.394	C_6_H_6_O_3_	126.11	49.43	17.96	Antioxidant, anti-inflammatory, antibacterial, antiproliferative	[52,53]
3-Deoxy-d-mannoic lactone	23.407	C_6_H_10_O_5_	162.14	6.27	1.73	Antibacterial, antimicrobial	[54]

## Discussion

Due to the modern lifestyle, urolithiasis continues to be a global medical burden and is getting worse every day and seems to have a very high recurrence rate. The present work aimed to evaluate ACV’s antioxidant and antiurolithiatic effect by DPPH assay, *in vitro* nucleation, crystallization, growth assays and GC–MS analysis. ACV exhibited dose-dependent radical scavenging activity against free radicals. DPPH Alkaloids, flavonoids, phenolic compounds, saponins, steroids, tannins and terpenoids can be found in novel sources through phytochemical screening, which is crucial for both industrial and therapeutic purposes [20]. The phytochemical screening of ACV disclosed the presence of constituents like flavonoids, polyphenols, etc., which have been proven against free radical scavenging and anticancer actions. Alkaloids, flavonoids, phenolic compounds, saponins, steroids, tannins and terpenoids can be found in novel sources through phytochemical screening, which is crucial for both industrial and therapeutic purposes [36]. Due to the increased efficacy, therapeutic value and concern over possible risks from existing medical therapy, complementary and alternative medicine, especially those utilizing herbs and medicinal plants, is one of the best treatments for various disorders [26]. The chemical compounds or ingredients in fermented fruit that have a clear physiological or biochemical effect on the human body give them their medicinal worth [42]. While endemic infantile bladder stone illness was widespread in vast parts of impoverished nations, upper urinary tract stone prevalence has recently increased in Western countries [43,44]. Dietary calcium, phytate and fluid consumption were all linked to a lower incidence of stone formation, especially in younger women [45]. While sugar and animal protein raised the likelihood of stone incidence. While magnesium, potassium and fluid intakes lowered the risk, and total vitamin C consumption raised the risk of symptomatic nephrolithiasis in older persons, there was no connection between dietary calcium and stone formation in this population [46,47]. It is well-established that plants with diuretic and antioxidant properties have inhibitory effects on the formation, nucleation and growth of crystals, rendering them antiurolithiatic [48]. Furthermore, kidney cell oxidative stress is one of the causes or effects of numerous diseases, including urinary stones. Therefore, the antioxidant capacity of ACV was also assessed. The ACV demonstrated a substantial suppression of the DPPH radical in the DPPH assay. The fractions demonstrated the ability to convert the stable DPPH radical (purple) to DPPH, which is not radical (yellow). At 60 mg/ml, the maximum percentage of inhibition was seen at 86.75 percent. In comparison, at 10 mg/ml, the smallest percentage of inhibition was seen at 46.14 percent-the half maximum inhibitory concentration (IC_50_ was found to be 54.7179). The ability of ACV to scavenge DPPH reactive oxygen species are produced when different crystal types, such as calcium phosphate, uric acid and CaOx, are exposed to renal epithelial cells where further reactive oxygen species contributes to the formation of kidney stones [40].

Experimental *in vitro* studies include nucleation, aggregation and growth assay for urolithiasis conditions. Nucleation assay reveals that crystal formation was inhibited at increasing concentration with an Ic_50_ value of 48.1646 μg/ml. This result agreed with previous experiments performed [35] and showed inhibition of nucleation at the highest concentration and found to be 61 ± 3.6%, respectively, for nucleation. Aggregation assay showed minimum absorbance in the highest concentration, indicating the reduction of stone formation. Furthermore, the Growth assay showed the highest inhibition of 81.59% in the increasing concentration with similar studies conducted on ACV and found to be 51.79%, respectively [41]. Several phytoconstituents discovered during preliminary screening and GC–MS that are pharmacologically active may be associated with the results of the antioxidant activity and *in vitro* inhibition. Different phytocompounds are known to affect metabolic processes in different ways, including 1,2-Cyclooctanedione, 2,4-Dihydroxy-2,5-dimethyl-3(2H) furan-3-one, and Bicyclo [2.2.1], heptane-2-carboxylic acid, 1,5-anhydro-6-deoxyhexo-2,3-diulose, 5-hydroxymethylfurfural and 3-deoxy-d-mannoic lactone. Further, identified bioactive compounds can be subjected to *in silico* molecular docking to identify their potential to bind to target receptors. All data paves the way for exploring the biological activity of identified compounds with affinity.

### Limitations of the study

- GC–MS was used to analyze ACV. GC–MS will only reveal the volatile constituents and will miss the nonvolatile phytochemicals. Thus, many critical phytochemical constituents will be missed. LC–MS technique could be done to probe further analysis.
- We compared mass spectra (MS) with the MS library entries of mass spectra databases rather than the linear retention index (LRI) technique.

## Conclusion

In Summary, ACV could be a potential source of a natural antioxidant drink based on *in vitro*, phytochemical and GC–MS analysis which was noteworthy and thus can be used as a therapeutic agent against bladder stones. The IC_50_ value of ACV in DPPH was found to be around 7 μg/ml and the IC_50_ value of the ACV in ABTS assay was around 9 μg/ml. The information serves as a foundation for evaluating the protective effects of plant-based therapies against free radical scavenging effects. The findings of this research support the therapeutic application of ACV. Furthermore, additional research is required to identify the crude substances by LC–MS analysis, characterize it and investigate the likely mechanism(s) underpinning the augmentation of urolithiasis activity. Because of the immense potential of ACV to treat a wide range of illnesses and disorders, the pharmacokinetic activity must be investigated. Additionally, the activity of discovered compounds can be docked with target receptors to find the compound’s affinity to the target site.

**Executive summary**

**Apple cider vinegar profile**

- Apple cider vinegar (ACV) is a wonder food with many medicinal properties that still need to be thoroughly explored.

***In* *vitro* studies**

- The results demonstrate that ACV can inhibit the nucleation, growth and aggregation of CaOx crystals.

**Management**

- ACV seems to be helpful to the current therapy in preventing kidney stones.

**Antioxidant status**

- ACV has better antioxidant activity, a critical factor for reducing free radical scavenging in the body.
